# Complications of electrosurgery: mechanisms and prevention strategies

**DOI:** 10.52054/FVVO.16.4.048

**Published:** 2024-12-27

**Authors:** S.M. El-Sayed, E Saridogan, M.M. El-Sayed

**Affiliations:** Orthopaedic Department, Southend University Hospital, Essex, SS0 0RY, UK; Obstetrics and Gynaecology Department, University College London Hospital, London, WC1E 6DB, UK; Obstetrics and Gynaecology Department, Darent Valley Hospital, Kent, DA2 8DA, UK

**Keywords:** Electrosurgery, complications, risks, hazards, surgical fires, electromagnetic interference

## Abstract

**Background:**

Electrosurgery is widely used in all surgical specialities. There is evidence that surgeons in different disciplines and with different experience levels have an inadequate understanding of the basics of electrosurgery and its complications. This can increase the risk of electrosurgical complications. Despite its improved safety technology, electrosurgery is still associated with serious morbidity and mortality. In addition, such adverse outcomes will incur financial losses to our health system due to the costs of repeated operations, prolonged hospital stays, and litigation.

**Objectives:**

To identify the various mechanisms of electrosurgical complications and to highlight the recommended actions to prevent such complications.

**Materials and Methods:**

Narrative review based on a literature search of the Medline database using the following search terms: “electrosurgery”, “complications”, “risks”, and “adverse effects”, with further citation searching for related articles.

**Main Outcome Measures and Results:**

The paper does not address specific research questions but addresses common knowledge gaps in the mechanisms of electrosurgical complications among surgeons.

**Conclusions:**

Electrosurgical devices can cause severe complications such as unintended tissue burns, surgical fires, smoke hazards, and interference with implantable devices. Although such energy devices are designed with increasingly improving safety features, an adequate understanding of the circumstances, mechanisms, and prevention of these complications by the surgical team is the cornerstone in mitigating such risks.

## Introduction

The use of cautery dates as far back as ancient times, with evidence of the use of heat to treat wounds. This set the precedent for the use of heat in therapeutic contexts, a key principle in electrosurgery. The therapeutic potential of electricity was established later in the early 20th century, with the likes of Franz Nagelshmidt using heat generated from electrical currents to treat a range of ailments; a method he termed diathermy (Massarweh et al., 2006). In 1926, biophysicist Bovie built upon these established principles to invent his electrosurgical apparatus, which was used by the neurosurgeon Cushing to remove vascular tumours once deemed inoperable. Their joint work demonstrated the superiority of Bovie’s electrosurgical apparatus in achieving good haemostasis with less infection and tissue damage. This revolutionised surgical practice and changed surgeons’ scepticism towards electrosurgery to widespread acceptance. Despite his early success with electrosurgery, Cushing reported an awful electric shock he had during one of his electrosurgical procedures as well as, on another occasion, a surgical fire (Carter, 2013). Despite the technological advances made since, there is evidence that knowledge gaps still exist among surgeons of different specialities and experience regarding their understanding of the basic principles and hazards of electrosurgery which can increase the chances of complications (Ha et al., 2018). This paper is written to plug these gaps and to promote the safe use of electrosurgical devices. This narrative review focuses purely on complications – for explanations of the various tissue effects, as well as monopolar and bipolar devices, we refer the reader to a paper written by Koninckx et al. (2024).

## Complications

The incidence of electrosurgical complications is about 2 – 5 /1000 procedures. They are mainly unintended tissue burns, which can have serious sequelae when affecting vital structures such as bowels, ureters, major blood vessels, and nerves (Martin et al., 2016). They also have financial implications due to medical malpractice claims (Sandberg et al., 2017). Certain electrosurgical complications are more common during laparoscopic surgery. Monopolar devices are associated with specific complications such as the historic alternate site and dispersive electrode burns, insulation failure, and direct, capacitive, and antenna coupling. Electrosurgical bowel perforations usually present on postoperative day 4 to day 10, depending on the degree of the coagulative necrosis, and can be fatal. Histopathology can differentiate bowel perforations caused by electrosurgery from perforations caused by a different pathology (Overbey et al., 2015). Electrosurgical complications also include smoke hazards, surgical fires, and electromagnetic interference (Table I). These complications are related to surgeons’ experience as well as their understanding of electrosurgery and the electrosurgical devices in use (Feldman et al., 2013). Adequate knowledge of the principles of electrosurgery, the mechanisms of its complications, as well as the operational instructions and troubleshooting of the various electrosurgical devices, would help minimise electrosurgical hazards and promote the safe use of such devices. Generally, surgeons have knowledge gaps in their understanding of electrosurgery (Fuchshuber et al., 2015). Our paper, which focuses on the various mechanisms of electrosurgical complications and how to prevent them complements a recent paper covering the biophysical principles of electrosurgery (Koninckx et al., 2024). Diagnosis and management of such complications are beyond the scope of this paper.

**Table I:** Electrosurgical complications.

### Unintended burns

These electrosurgical burns can affect the patient or the surgical team. When affecting the patient, they can be skin or internal burns, with the latter generally being more serious. There are several mechanisms that can produce such burns.

#### Lateral thermal spread

Lateral thermal spread is the unintended heat transfer from an applied energy device into nearby tissues, potentially causing collateral damage. This is the most common mechanism of tissue injury when using electrosurgery, especially close to vital structures. Surgeons need to be aware that the risk of this complication increases with higher power and voltage as well as longer activation times. With traditional bipolar instruments, surgeons must rely on visual cues to avoid unnecessarily long activation times; namely the cessation of water vapour (bubbles) release from the treated tissues. This signals that the desired tissue effect of desiccation has been achieved. These bubbles create increased impedance and can stop a current with low voltage (< 200 V). On the other hand, prolonged activation with higher voltage current (>200 V) causes excessive tissue effect (carbonisation) and increased lateral thermal spread as such current flow is not stopped by the released water bubbles. To prevent this, therefore, surgeons should deactivate the device at the end of the vapour phase. Monopolar coagulation results in more thermal spread than bipolar, with ultrasonic causing the least thermal spread (Sutton et al., 2010). The pure-cut waveform (non-contact) also produces negligible lateral thermal spread. Inadvertent contact of energy devices with nontargeted tissues can occur when the anatomy is distorted or when the tissues are very close to each other (Vilos, 2018). Where vital structures lie very close to the target tissue, the use of alternative haemostatic techniques, such as clips, staples, and ligatures, is recommended instead of electrosurgery.

Surgeons should avoid contact between the tips of various advanced bipolar sealers and vital structures, as heat can be transferred at the backs of their tips (Suzuki et al., 2019).

#### Lateral thermal spread

Unintended activation of energy devices while touching tissues can result in serious injury depending on the touched tissue. To prevent this risk, the loud activation tone should be on, and the surgeon should be the one to activate the device. It is safe practice to keep the energy device in a dry holder outside the patient when not in use.

#### Residual heat

After deactivation, surgical energy devices cool down at different rates, with ultrasonic devices having the most prolonged residual heat, followed by monopolar, then bipolar, with argon beam coagulators having the least residual heat (Govekar et al., 2011). The differences in cooling rates between these devices lie mainly in the different ways in which they generate heat. The material composition of the instruments also plays a role – materials with higher heat capacities, such as stainless steel, tend to retain heat for longer and have slower cooling rates.

Ultrasonic devices generate heat through mechanical vibration, leading to prolonged residual heat and slow cooling rates of the thermal tip. Although both monopolar and bipolar instruments use electrical energy, the latter has a faster cooling rate than the former, as they confine the current between two electrodes, resulting in more localised heating and faster cooling compared to monopolar instruments. Argon beam coagulators have the fastest cooling rate, due to the flow of Argon gas, which aids in rapid heat dissipation.

It is good practice not to manipulate vital structures with these devices after deactivation to avoid unintended tissue burns. After deactivation, surgeons can cool down such devices by contacting the omentum or irrigation fluid or waiting for 30 seconds. The former is possible as any damage done to the omentum by cooling the instrument bears little consequence for the patient.

If you suspect bowel injury because of this mechanism, inspect the bowel thoroughly and suture any blanched area to prevent postoperative bowel perforation due to coagulative necrosis (Jones et al., 2015).

#### Pedicle effect (funnelling)

**Figure 1:**
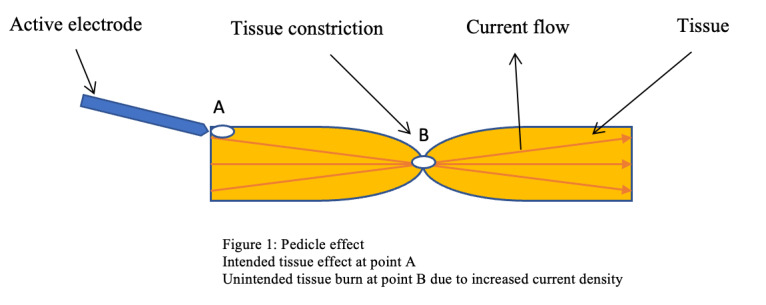
Pedicle effect; Intended tissue effect at point A; Unintended tissue burn at point B due to increased current density.

These unintended tissue burns occur at constricted points along the current pathway away from the contact point of the monopolar device. Higher current density at these constriction points explains this injury. Examples of such constricted areas include ducts, pedicles, and adhesion bands (Figure 1). The use of monopolar devices to dissect the cystic duct during laparoscopic cholecystectomy is not recommended as it can cause a burn in the bile duct due to increased current density at its narrowest point. This can then lead to delayed pinhole perforation postoperatively (Humes and Ahmed, 2010).

#### Insulation failure

This mechanism occurs when the insulation layer of the active electrode is breached, leading to electric current leakage. It works as a second active electrode, delivering 100% of the energy used to any tissue touching the breach point. It is more common in reusable laparoscopic instruments (20%) due to the damage caused by repeated use, cleaning and sterilisation (Espada et al., 2011). It also affects disposable instruments (3%) when a higher voltage overcomes the dielectric strength (maximum voltage required to produce a breakdown of the insulating layer) of the used instrument (Montero et al., 2010). Therefore, keeping the voltage lower than the dielectric strength of the instrument in hand is recommended to avoid intraoperative insulation failure. The dielectric strength can be found on the instrument or in its instruction manual. In laparoscopic instruments, the distal third is the most affected site for insulation defects. Smaller defects are associated with a higher current density and more thermal tissue injury compared to larger ones. Burns of the lower genital tract have been reported due to insulation failure of monopolar hysteroscopic instruments (Vilos et al., 1997).

Although routine visual inspection of instruments preoperatively is common practice, studies suggest this may be inadequate due to the presence of microscopic, invisible defects which may be missed. As a result, some now recommend the use of other, more technologically advanced testing methods, such as insulation failure detectors (Tixier et al., 2016).

**Figure 2:**
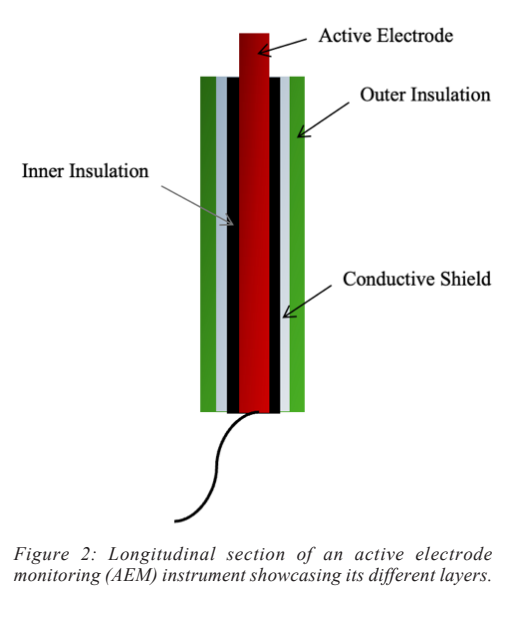
Longitudinal section of an active electrode monitoring (AEM) instrument showcasing its different layers.

Active electrode monitoring (AEM) technology was developed to eliminate insulation failure and largely minimise capacitive coupling in laparoscopic instruments (Vancaillie, 1998). The AEM instrument is different from its regular monopolar counterpart in that it has an extra conductive shield with an outer insulation layer (Figure 2). The AEM circuit allows the conductive shield to work as a second dispersive electrode, draining any current resulting from insulation failure or capacitive coupling back to the generator. It deactivates the generator once dangerous levels of such stray currents are found. It works with the split dispersive electrode but not with the capacitive one.

#### Direct Coupling

Direct coupling occurs when an activated electrode contacts and energises another metal instrument, such as a laparoscope or a probe. If the energised second instrument is in contact with a tissue, unintended thermal burn might develop away from the surgeon’s view. It is to be noted that 100% of the energy used can pass to the second instrument. The common surgical practice of buzzing the haemostat is an intentional direct coupling for haemostasis (Jones et al., 2015). Although more likely to occur in monopolar systems, it can also occur in bipolar systems (Gentles, 2006).

To minimise the risk of harmful direct coupling, the surgeon should always be aware of the active electrode’s position and not activate it until its tip is visible. They should also endeavour to avoid contact between the metal cannula and the bowel. Also, surgeons are advised against the use of monopolar devices for haemostasis of a bleeding staple line.

#### Capacitive Coupling

On the other hand, capacitive coupling occurs when an unintended capacitor is formed between an active electrode and its intact insulation and a nearby conducting material, such as a metal instrument or body tissue. In this arrangement, the active electrode induces a current in the other conductor through its intact insulation, which can cause unintended burns.

**Figure 3:**
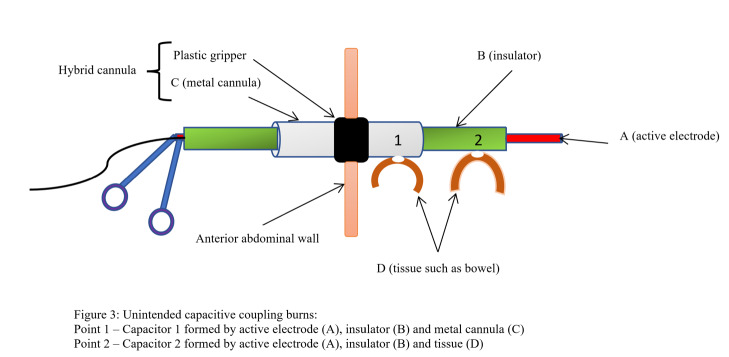
Unintended capacitive coupling burns: Point 1 – Capacitor 1 formed by active electrode (A), insulator (B) and metal cannula (C). Point 2 - Capacitor 2 formed by active electrode (A), insulator (B) and tissue (D).

Examples of this mechanism are shown in Figure 3. Unlike direct coupling and insulation failure, only a fraction of the used electricity is transferred (Odell, 2013). Such a fraction is dependent on the distance between the two conductors, the insulation in between, the voltage, and the activation time (Vilos et al., 2001). In bipolar devices, the lower voltage used, and the closeness of the two electrodes almost removes the risk of alternate site burns as well as burns from direct coupling (Livaditis, 2001). Moreover, capacitive coupling does not occur with bipolar devices as the current with resulting corona discharge travels along the wires in opposite directions leading to the corona discharge cancelling itself out (Vilos and Rajakumar, 2013).

Capacitive coupling is more likely to cause harm if hybrid cannulas are used (Figure 3). Though mainly an issue in laparoscopic surgery, it can also occur in both hysteroscopic and open procedures (Vilos, et al., 2006).

By understanding the mechanism underpinning capacitive coupling, surgeons can reduce its risks by avoiding instrument arrangements that could form potentially harmful capacitors. Using the lowest effective power setting for the shortest possible time, opting for Cut rather than Coag waveform for coagulation, and avoiding open activation can also lower the risk of harmful capacitive coupling (Robinson et al., 2010)

Improved technological systems, such as active electrode monitoring and instant response, can also mitigate the harmful effects of capacitive coupling. The use of alternative energy devices, such as bipolar and ultrasonic devices, eliminates the specific risks of monopolar devices (Vancaillie, 1998).

With the recent resurgence of single-port laparoscopy, it is important to note that it increases the risks of direct and capacitive coupling of monopolar devices due to the proximity and crossing of laparoscopic instruments (Abu-Rafea et al., 2011).

#### Antenna Coupling

This recently described mechanism happens when the active electrode (transmitting antenna) emits electromagnetic energy (waves) that propagate through the air and are captured by nearby inactive conductors (receiving antenna) (Robinson et al., 2012). Transmitting antennas are formed when alternating current flowing through a wire generates electromagnetic waves of the same frequency. The receiving antenna can be the wires of the camera cord, neuromonitoring leads (Parikh et al., 2003), and electrocardiography pads (Aigner et al., 1997). The energy transferred raises the receiving antenna temperature, potentially causing burns should it contact the patient. Antenna coupling is like capacitive coupling insofar as there is energy transfer through intact insulation and without direct contact between instruments.

The antenna coupling risk increases when the cords (wires) are long, close, and parallel to each other (Jones et al., 2015). The risk can be lowered by keeping cords and wires separate from each other and avoiding their parallel arrangement. This also applies to the cord of the dispersive electrode, which can act as a transmitting antenna (Townsend et al., 2016). Using the lowest effective power setting and separating the laparoscopic stack from the electrosurgical unit also helps reduce the risk, as does using the cut waveform rather than the coag waveform for coagulation. Additionally, opting for bipolar or ultrasonic devices, which remove the risk of antenna coupling altogether, is advisable (Townsend et al., 2015).

#### Alternate site burns

Early generators were designed to allow the current to eventually sink into the ground (grounded generators). In case of increased impedance to current flow, it seeks an alternative path of least resistance to the ground. This path can be any conductor touching the patient and ground at the same time, such as intravenous poles, ECG leads and the theatre table. As the contact areas with such objects are narrow, current density will increase, leading to deep burns at such contact points. As technology improved, isolated generators were developed to address this issue (Lipscomb and Givens, 2010). It has nearly nullified these burns, with only rare cases now reported (Sultan et al., 2020).

#### Dispersive electrode burns

In monopolar circuits, current from the generator is concentrated at the tip of the monopolar device to produce the intended effect, and then passes through the patient to return to the generator through the wide return electrode, with a low current density. To maintain the low current density at the dispersive electrode, it should be applied over an even, well- vascularised muscle mass near the operative field with less chance of fluid accumulation. Partial detachment or improper application of the return electrode increases current density and, therefore, its associated risk of dispersive electrode burns. Surgeons should avoid locating the dispersive electrodes over scars, hair, bony prominences, metal prostheses, tattoos, or pressure points to minimise such risk. These burns are different from chemical burns and pressure sores as they are instant, smaller than the dispersive electrode, and located beneath it.

After the wide application of contact quality monitoring (CQM) technology in the 1980s, dispersive electrode burns rarely occur nowadays (Vilos, 2018). The CQM circuit detects any increased impedance due to partial detachment of the split dispersive electrode and automatically stops the electrosurgical unit (Odell, 2013). In electrosurgical procedures, when higher currents or extended device activations are expected, the use of two dispersive electrodes is recommended (Vilos, 2018). The design of the large capacitive dispersive electrode avoids the need for CQM with nearly no risk for dispersive electrode burns. Yet, an unintended burn was reported when a stainless tube tree was placed on the capacitive pad (Park et al., 2014).

Tables II and III summarise the key points for the safe use of monopolar and bipolar devices, respectively.

**Table II:** Golden rules for the safe use of monopolar devices.

**Table III:** Golden rules for the safe use of bipolar devices.

#### Surgical gloves

Although surgical gloves are used mainly to prevent infection transmission among patients and the surgical team, they can contribute to incidental hand burns and electric shocks to the latter during electrosurgical procedures. Three mechanisms have been cited as explanations for these burns and shocks (Tucker and Ferguson, 1991). Firstly, while holding an activated instrument with a gloved hand, capacitive coupling can induce electric current into the surgeon’s hand through the intact glove. Such induced current increases with a smaller contact area, thinner gloves, and higher voltage and power settings. Secondly, during electrosurgical sparking, partial rectification of the alternating current produces a direct current, which can overcome the low resistance of the wet glove to cause electric shock and hand burns. Thirdly, the use of high-voltage settings, as in fulguration and open activation, results in glove dielectric breakdown (perforation) with a resultant hand burn and electric shock. To avoid such risks, surgeons are advised to double glove, use the lowest effective power setting, and avoid prolonged or open activation of electrosurgical devices. Although such injuries are linked mainly to monopolar devices, a defective bipolar device was reported to cause similar injuries to the anaesthetist on touching the patient (Gilbert et al., 1992).

### Surgical smoke

**Figure 4:**
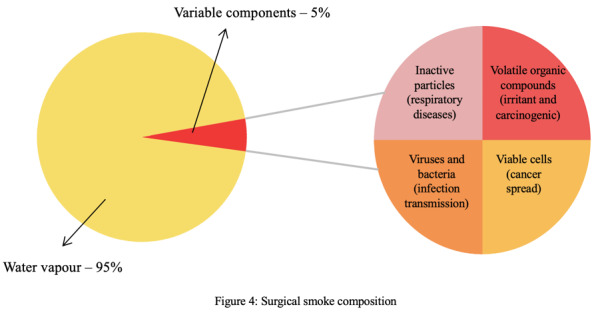
Surgical smoke composition.

Surgical smoke is an aerosol by-product generated when different energy devices are used to treat tissues during surgery. It is formed mainly of water vapour (95%), with the remaining 5% consisting of inert particles, chemicals, viable cells, and microorganisms (Figure 4). Its components will vary depending on the type of tissue treated and the energy device used. Electrosurgery produces smaller particles in surgical smoke, whereas ultrasonic energy is associated with more viable cells and microorganisms, which can be attributed to the lower temperature generated by ultrasonic devices. It is the 5% fraction of the surgical smoke that poses health hazards to the patient and surgical team. One of the common hazards of surgical smoke is reduced surgical visibility, which can lead to surgical complications. Monopolar instruments produce more smoke with less visibility compared to their bipolar and ultrasonic counterparts (Weld et al., 2007). Generally, surgical smoke has a bad smell and can compromise the air quality in the theatre, which can affect theatre staff.

The inactive smoke particles are deposited at different levels of the respiratory tract depending on their size. Bigger particles of 5 μm or more are trapped in the respiratory tract down to the bronchi, which can explain the higher prevalence of upper respiratory conditions, asthma, and bronchitis among scrub nurses. On the other hand, smaller particles of a size less than 2 μm can reach the bronchioles and alveoli, causing chronic lung diseases (Liu et al., 2019). Although surgical smoke contains many carcinogenic organic compounds, lung cancer incidence is not higher in theatre staff compared to the general population (Gates et al., 2007).

As surgical smoke can carry viable viruses and bacteria, it can present a significant risk of infection transmission to the surgical team (Kwak et al. 2016). Not only may surgical smoke transmit viruses and bacteria, but it also has the potential of transmitting viable cancer cells. The cancer risk of surgical smoke may be related to its carcinogenic compounds. Additionally, viable cancer cells within the smoke can cause laparoscopic port metastasis (chimney effect) away from the port used for tumour extraction. Reported cases of HPV tonsil cancer in surgeons were thought to be related to their years of smoke exposure while treating HPV-related cervical and vulvar lesions (Liu et al., 2019).

Most surgeons are not aware of the health hazards associated with surgical smoke. Therefore, enrolling all theatre team members in relevant training programs would increase their awareness of such hazards and the appropriate protective measures (Steege et al., 2016). On using electrosurgery, surgeons are advised to avoid tissue charring to reduce surgical smoke production (El- Sayed and Saridogan, 2021). Surgical masks are not effective in filtering particles less than 5 μ, including inert particles, pathogens, and volatile organic compounds. Therefore, high-filtration masks such as N95 and masks containing activated carbon are recommended for better protection (Lewin et al., 2011). In addition, appropriate ventilation systems within theatre and the use of smoke extractors during electrosurgery would minimise surgical smoke risks (Zhou et al., 2023).

### Explosions

**Figure 5:**
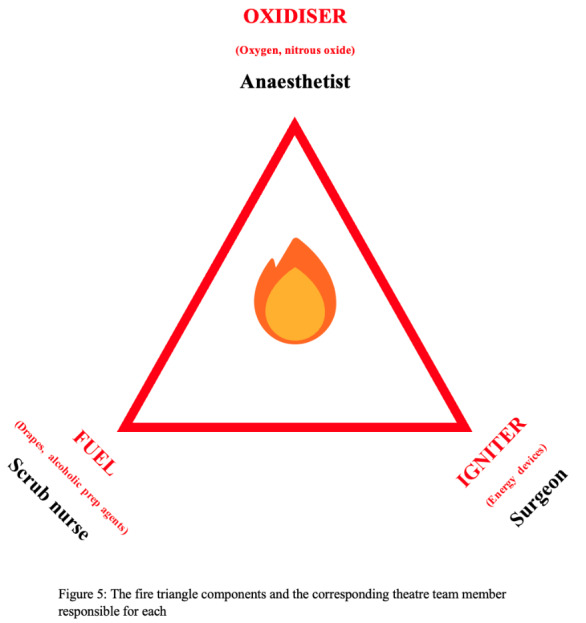
The fire triangle components and the corresponding theatre team member responsible for each.

While surgical explosions are uncommon, they can pose a significant risk to life. Similar to fires, explosions require the three elements of the fire triangle to be present (Figure 5).

Flammable gases such as hydrogen and methane are generated in the gastrointestinal tract via bacterial metabolism, potentially leading to deadly explosions during colonoscopic electrosurgery (Ladas et al., 2007). Crucially, more than 5% oxygen is necessary for an explosion to occur (Macdonald, 1994). Early reports concerning explosions during colonoscopic electrosurgery recommended complete bowel preparation prior to colonoscopy to minimise the presence of these flammable gases, alongside the use of carbon dioxide as a distension medium to mitigate combustion. Endoscopists are advised against the use of mannitol and sorbitol for pre-colonoscopy bowel preparation since their fermentation by bacteria produces explosive gases.

There have been documented instances of grave explosions when using nitrous oxide, oxygen, or a combination of oxygen and carbon dioxide to create pneumoperitoneum during laparoscopic electrosurgery. As an oxidiser, nitrous oxide can provide the oxygen needed for explosions and fires. Nowadays, nitrous oxide is not used in general anaesthesia as it may spread out into the peritoneal cavity and combine with the carbon dioxide used in laparoscopic pneumoperitoneum at significant levels. If bowel perforation occurs, flammable gases released from the bowel can create an explosion risk during electrosurgery. To minimise such risks, 100% carbon dioxide is used to create pneumoperitoneum, as it is soluble in blood, easily expelled via the lungs, and non- combustible (Neuman et al., 1993).

As previously stated, explosive gases can accumulate in the gastrointestinal tract, particularly in cases of obstructed hiatus hernias pyloric stenosis, or bowel obstruction. Under such circumstances, explosions have been reported whilst using electrosurgical devices to open the stomach or bowel. In instances of gastric or bowel perforations, these explosive gases can escape into the peritoneal cavity, posing an explosion risk when using electrosurgical devices to access the peritoneum. To prevent such explosions, the use of a cold knife rather than electrosurgery is recommended to open the stomach, bowel, or peritoneum if obstruction and/or perforation are suspected. Also, over-oxygenation preoperatively is discouraged in these situations (Mumith et al., 2013).

During electrosurgical transurethral resection of the prostate or bladder polyps, hydrogen and other gases are released into the bladder. Such a flammable gas mixture, combined with electrosurgery and atmospheric oxygen introduced into the bladder during the procedure, can result in an explosion and bladder rupture. Strategies like repeated bladder washouts, applying suprapubic pressure, and utilising a suprapubic shunt can aid in removing gas from the bladder (Ning et al., 1975).

### Surgical Fires

Surgical fires are fires that affect the patient during surgery. Despite being rare, these fires can result in serious injuries to both patients and healthcare professionals. In addition, they can attract high compensation costs to healthcare providers (Mehta et al., 2013). Due to under-reporting, the incidence of surgical fires is usually underestimated. The fire triad is formed of an oxidiser, an igniter and fuel with a different member of the theatre team responsible for each of the three components (Figure 5). The coexistence of the three components can start fires, with the oxidiser (high- concentration oxygen) being the most important factor. Preventing this risky combination can eliminate surgical fires.

#### Prevention of surgical fires

Understanding how the three components of the fire triad interact to cause surgical fires is crucial for fire prevention and control. Carrying out a fire risk assessment before every surgical procedure with effective communication among the theatre team is recommended for fire prevention (Yardley and Donaldson, 2010). The risk factors to be assessed include whether the surgery is above the xiphisternum, the use of open oxygen, energy devices, and alcoholic skin prep. Based on these four factors, fire risk is categorised into low-risk, intermediate-risk or high-risk. Accordingly, a plan with designated roles is agreed upon. Non-alcoholic antiseptics are preferred to the alcoholic ones. The latter, when used, needs time to dry completely and should be kept away from the patient’s hair (Jones et al., 2017).

To enhance fire safety during procedures, several key measures should be implemented. First, it is advisable to use a closed oxygen system instead of an open one when appropriate. Additionally, oxygen should not be allowed to accumulate beneath tented drapes, as this poses a significant risk. It is also important to avoid using surgical energy devices in high-oxygen environments, particularly for surgeries involving the head, neck, and chest. If the use of these devices is necessary, ensure adequate time is given for the room’s oxygen levels to fall after the oxygen supply is lowered.

Moreover, energy devices should not be used to enter the trachea during tracheostomy procedures. Finally, energy devices should be handled safely by storing them securely in a holster when not in use, rather than placed on drapes or directly on the patient. Implementing these measures is essential for surgical fire prevention.

#### Management of surgical fires

Due to the rare occurrence of surgical fires, all theatre team members should regularly undergo mandatory simulation training in fire management. Early identification and extinguishing of surgical fires will significantly reduce their adverse impact. Early signs of a potential fire include heat, smoke, flame or flash, unusual sounds, odours, and the discolouration of drapes or the breathing circuit (Jones et al., 2019). Once discovered, a fire should be declared, the fire alarm activated, and surgery stopped. Tapping with the gloved hand or using water, saline or wet towels may be enough for small fires not involving the airway. Drapes should be removed to assess underneath for any further fires. For persistent and larger fires, burning drapes should be removed from the patient and the fire put out by a carbon dioxide extinguisher. At the same time, the oxygen should be stopped, and ventilation maintained with a self-inflating bag (Apfelbaum et al., 2013). For airway fires, the endotracheal tube is removed, oxygen is stopped, and fire is extinguished using saline poured down the airway. The patient is then cared for in a safe area where air ventilation is established after reintubation. Additionally, any patient burns are assessed and managed accordingly (Day et al., 2018).

### Electromagnetic interference with other devices

Electrosurgical devices, particularly monopolar ones, emit electromagnetic waves that can affect the functionality of other nearby devices – a phenomenon known as electromagnetic interference (EMI).

#### Implantable electronic devices

The use of implantable electronic devices is on the rise, making it not uncommon to operate on patients with them. Patients with cardiac implantable electronic devices (CIED) such as pacemakers, implantable cardioverter defibrillators (ICD), cardiac resynchronisation devices, and ventricular assist devices are high-risk surgical patients due to the potentially serious effects of electrosurgery on such devices. EMI can cause dysfunction or damage to these devices. As such, during electrosurgery, these patients may develop hypotension, arrhythmias, and asystole (Meyer and Crew, 2008). The EMI depends on device type and setting, power setting, voltage, and distance of the generator to the implanted device, as well as the surgical site (above or below the umbilicus). The clinical effect of EMI will depend largely on the degree of patient dependency on their CIED.

Surgery below the umbilicus carries a negligible risk of EMI compared to surgery above the umbilicus (Schulman et al., 2019). The intraoperative management of monopolar devices to minimise EMI includes distant placement of the dispersive electrode and generator from CIED, use of the lowest effective power and voltage, and avoiding passing the active electrode over the CIED. For patients at high risk of EMI, it is recommended to use alternative energy devices such as bipolar or ultrasonic devices (Bentham and Preshaw, 2021).

The electrosurgical interference with other implanted devices such as neurostimulators, cochlear implants, and infusion pumps is not immediately life-threatening as with CIED. Nonetheless, it remains very important to have these patients assessed preoperatively by a multidisciplinary team of a cardiologist, surgeon, anaesthetist, and theatre nurse and to formulate a detailed preoperative, intraoperative, and postoperative management plan.

#### Electrocardiogram (ECG)

Electromagnetic interference can cause ECG artefacts, mostly during arcing. Increasing the distance between ECG leads and the generator, using the lowest effective power setting, and the avoidance of prolonged or open activation can aid in reducing this effect.

#### Video Imaging System

The high-frequency current of electrosurgery emits electromagnetic waves around the wires of the electrosurgical circuit. These waves can interfere with the laparoscopic video imaging systems as they have a similar wavelength. This interference may manifest as noise spots, loss of video synchronisation, or complete system failure. The interference becomes more pronounced with higher power settings, voltage, and arcing and when the generator is close to the video system. EMI can be reduced by increasing the distance between the electrosurgical generator and the video system. Modern generators have better control of power output with less EMI. Moreover, newer video systems are less affected by EMI as they have electromagnetic shielding. (El-Sayed and Saridogan, 2021).

## Conclusion

Electrosurgery has revolutionised modern surgical practice, emerging as the most widely utilised energy modality across various disciplines. While it has contributed to improved surgical outcomes, it is also associated with potentially fatal complications. Despite ongoing advancements in the technological design of electrosurgical devices aimed at enhancing safety, adverse events continue to occur, often due to improper usage. Evidence suggests that surgeons’ understanding of the principles and complications associated with electrosurgery remains inadequate. Therefore, it is essential for the surgical team to fully comprehend the operational aspects of electrosurgery, the circumstances and mechanisms underlying its complications, and effective strategies for their prevention.

## References

[1] Abu-Rafea B, Vilos GA, Al-Obeed O (2011). Monopolar electrosurgery through single-port laparoscopy: a potential hidden hazard for bowel burns.. J Minim Invasive Gynecol.

[2] Aigner N, Fialka C, Fritz A (1997). Complications in the use of diathermy.. Burns.

[3] Apfelbaum JL, Caplan RA, Barker SJ (2013). Practice advisory for the prevention and management of operating room fires: an updated report by the American Society of Anesthesiologists Task Force on Operating Room Fires.. Anesthesiology.

[4] Bentham GL, Preshaw J (2021). Review of advanced energy devices for the minimal access gynaecologist.. TOG.

[5] Carter PL (2013). The life and legacy of William T. Bovie. Am J Surg.

[6] Day AT, Rivera E, Farlow JL (2018). Surgical fires in otolaryngology: a systematic and narrative review.. Otolaryngol Head Neck Surg.

[7] El-Sayed MM, Saridogan E (2021). Principles and safe use of electrosurgery in minimally invasive surgery.. Gynecol Pelvic Med.

[8] Espada M, Munoz R, Noble BN (2011). Insulation failure in robotic and laparoscopic instrumentation: a prospective evaluation. Am J Obstet Gynecol.

[9] Feldman LS, Brunt LM, Fuchshuber P (2013). Rationale for the fundamental use of surgical Energy™ (FUSE) curriculum assessment: focus on safety.. Surg Endosc.

[10] Fuchshuber PR, Robinson TN, Feldman LS (2015). Fundamental use of surgical energy (FUSE): Closing a gap in medical education.. Ann Surg.

[11] Gates MA, Feskanich D, Speizer FE (2007). Operating room nursing and lung cancer risk in a cohort of female registered nurses.. Scan J Work Environ Health.

[12] Gentles B (2006). Electrosurgical burn injuries in minimally invasive surgery.. CMBES Proc.

[13] Gilbert TB, Shaffer M, Matthews M (1991). Electrical shock by dislodged spark gap in bipolar electrosurgical device.. Anesth Analg.

[14] Govekar HR, Robinson TN, Stiegmann GV (2011). Residual heat of laparoscopic energy devices: how long must the surgeon wait to touch additional tissue?. Surg Endosc.

[15] Ha A, Richards C, Criman E (2018). The safe use of surgical energy devices by surgeons may be overestimated.. Surg Endosc.

[16] Humes DJ, Ahmed I, Lobo DN (2010). The pedicle effect and direct coupling.. Arch Surg.

[17] Jones DB, Brunt LM, Feldman LS (2015). Safe energy use in the operating room.. Curr Probl Surg.

[18] Jones EL, Overbey DM, Chapman BC (2017). Operating room fires and surgical skin preparation.. J Am Coll Surg.

[19] Jones TS, Black IH, Robinson TN (2019). Operating room fires.. Anesthesiology.

[20] Koninckx PR, Ussia A, Amro B (2024). Electrosurgery: heating, sparking and electrical arcs.. Facts Views Vis Obgyn.

[21] Kwak HD, Kim SH, Seo YS (2016). Detecting hepatitis B virus in surgical smoke emitted during laparoscopic surgery.. Occup Environ Med.

[22] Ladas SD, Karamanolis G, Ben-Soussan E (2007). Colonic gas explosion during therapeutic colonoscopy with electrocautery.. World J Gastroenterol.

[23] Lewin JM, Brauer JA, Ostad A (2011). Surgical smoke and the dermatologist.. J Am Acad Dermatol.

[24] Lipscomb GH, Givens VM (2010). Preventing electrosurgical energy-related injuries.. Obstet Gynecol Clin North Am.

[25] Liu Y, Song Y, Hu X (2019). Awareness of surgical smoke hazards and enhancement of surgical smoke prevention among gynecologists.. J Cancer.

[26] Livaditis GJ (2001). Comparison of monopolar and bipolar electrosurgical modes for restorative dentistry: a review of the literature.. J Prosthet Dent.

[27] Macdonald AG (1994). A brief historical review of non-anaesthetic causes of fires and explosions in the operating room.. Br J Anaesth.

[28] Martin KE, Moore CM, Tucker R (2016). Quantifying inadvertent thermal bowel injury from the monopolar instrument.. Surg Endosc.

[29] Massarweh NN, Cosgriff N, Slakey DP (2006). Electrosurgery: history, principles, and current and future uses.. J Am Coll Surg.

[30] Mehta SP, Bhananker SM, Posner KL (2013). Operating room fires: a closed claims analysis.. Anesthesiology.

[31] Meyer JP, Crew J (2008). The risks of diathermy in the urological patient with a pacemaker or an automatic internal cardiac defibrillator.. BJU Int.

[32] Montero PN, Robinson TN, Weaver JS (2010). Insulation failure in laparoscopic instruments.. Surg Endosc.

[33] Mumith A, Thuraisingham J, Gurunathan-Mani S (2013). Ignition of free gas in the peritoneal cavity: An explosive complication.. Case Rep Surg.

[34] Neuman GG, Sidebotham G, Negoianu E (1993). Laparoscopy explosion hazards with nitrous oxide.. Anesthesiology.

[35] Ning TC, Atkins DM, Murphy RC (1975). Bladder explosions during transurethral surgery.. J Urol.

[36] Odell RC (2013). Surgical complications specific to monopolar electrosurgical energy: engineering changes that have made electrosurgery safer.. J Minim Invasive Gynecol.

[37] Overbey DM, Townsend NT, Chapman BC (2015). Surgical energy-based device injuries and fatalities reported to the Food and Drug Administration. J Am Coll Surg.

[38] Parikh SN, Mehlman CT, Keith RW (2003). A third-degree burn caused by a neurogenic motor-evoked potential monitoring electrode during spinal surgery: a case report.. Spine (Phila Pa 1976).

[39] Park SS, Lim JA, Yeo JS (2014). Intraoperative electrical burn caused by stainless tube tree with noncontact electrosurgical ground.. Anesth Pain Med.

[40] Robinson TN, Pavlovsky KR, Looney H (2010). Surgeon-controlled Factors That Reduce Monopolar Electrosurgery Capacitive Coupling During Laparoscopy.. Surg Laparosc Endosc Percutan Tech.

[41] Robinson TN, Barnes KS, Govekar HR (2012). Antenna coupling-a novel mechanism of radiofrequency electrosurgery complication: practical implications.. Ann Surg.

[42] Sandberg EM, Bordewijk EM, Klemann D (2017). Medical malpractice claims in laparoscopic gynecologic surgery: a Dutch overview of 20 years.. Surg Endosc.

[43] Schulman PM, Treggiari MM, Yanez ND (2019). Electromagnetic Interference with Protocolized Electrosurgery Dispersive Electrode Positioning in Patients with Implantable Cardioverter Defibrillators.. Anesthesiology.

[44] Steege AL, Boiano JM, Sweeney MH (2016). Secondhand smoke in the operating room? Precautionary practices lacking for surgical smoke.. Am J Ind Med.

[45] Sultan SA, Alahmadi B, Mohabbat Sr A (2020). Hand Skin Burn as a Complication of Electrosurgery Use in Prone Position in Surgery: A Case Report.. Cureus.

[46] Sutton PA, Awad S, Perkins AC (2010). Comparison of lateral thermal spread using monopolar and bipolar diathermy, the Harmonic Scalpel™ and the Ligasure™.. Br J Surg.

[47] Suzuki T, Hattori R, Minagawa T (2019). Intestinal injury by heat conduction from surgical sealing devices. JSLS.

[48] Tixier F, Garçon M, Rochefort F (2016). Insulation failure in electrosurgery instrumentation: a prospective evaluation.. Surg Endos.

[49] Townsend NT, Nadlonek NA, Jones EL (2016). Unintended stray energy from monopolar instruments: beware the dispersive electrode cord.. Surg Endosc.

[50] Townsend NT, Jones EL, Paniccia A (2015). Antenna coupling explains unintended thermal injury caused by common operating room monitoring devices.. Surg Laparosc Endosc Percutan Tech.

[51] Tucker RD, Ferguson S (1991). Do surgical gloves protect staff during electrosurgical procedures?. Surgery.

[52] Vancaillie TG (1998). Active electrode monitoring. How to prevent unintentional thermal injury associated with monopolar electrosurgery at laparoscopy. Surg Endosc.

[53] Vilos GA, D’Souza I, Huband D (1997). Genital tract burns during rollerball endometrial coagulation.. J Am Assoc Gynercol Laparosc.

[54] Vilos G, Latendresse K, Gan BS (2001). Electrophysical properties of electrosurgery and capacitive induced current.. Am J Surg.

[55] Vilos GA, Newton DW, Odell RC (2006). Characterisation and mitigation of stray radiofrequency currents during monopolar resectoscopic electrosurgery.. J Minim Invasive Gynecol.

[56] Vilos GA, Rajakumar C (2013). Electrosurgical generators and monopolar and bipolar electrosurgery.. J Minim Invasive Gynecol.

[57] Vilos GA (2018). Understanding and practicing safe electrosurgery in the operating room.. J Obstet Gynaecol Can.

[58] Weld KJ, Dryer S, Ames CD (2007). Analysis of surgical smoke produced by various energy-based instruments and effect on laparoscopic visibility.. J Endourol.

[59] Yardley IE, Donaldson LJ (2010). Surgical fires, a clear and present danger.. Surgeon.

[60] Zhou YZ, Wang CQ, Zhou MH (2023). Surgical smoke: A hidden killer in the operating room.. Asian J Surg.

